# The Executive Functions in Overweight and Obesity: A Systematic Review of Neuropsychological Cross-Sectional and Longitudinal Studies

**DOI:** 10.3389/fpsyg.2019.02126

**Published:** 2019-09-20

**Authors:** Francesca Favieri, Giuseppe Forte, Maria Casagrande

**Affiliations:** ^1^Department of Psychology, “Sapienza” University of Rome, Rome, Italy; ^2^Department of Dynamic and Clinical Psychology, “Sapienza” University of Rome, Rome, Italy

**Keywords:** executive functions, obesity, overweight, cross-sectional studies, longitudinal studies

## Abstract

**Background:** The increasing incidence of people affected by overweight or obesity is a significant health problem. The knowledge of the factors which influences the inappropriate eating behaviors causing excessive body fat is an essential goal for the research. Overweight and obesity are significant risk factors for many health diseases, such as cardiovascular problems, diabetes. Recently, many studies have focused on the relationship between body weight and cognitive processes.

**Objectives:** This systematic review is aimed to investigate the existence and the nature of the relationship between excessive body weight (overweight/obesity) and executive functions, analyzing cross-sectional, and longitudinal studies in order to verify the evidence of a possible causality between these variables.

**Methods:** The review was carried out according to the PRISMA-Statement, through systematic searches in the scientific databases PubMed, Medline, PsychInfo, and PsycArticles. The studies selected examined performance on executive tasks by participants with overweight or obesity, aged between 5 and 70 years. Studies examining eating disorders or obesity resulting from other medical problems were excluded. Furthermore, the results of studies using a cross-sectional design and those using a longitudinal one were separately investigated.

**Results:** Sixty-three cross-sectional studies and twenty-eight longitudinal studies that met our inclusion and exclusion criteria were analyzed. The results confirmed the presence of a relation between executive functions and overweight/obesity, although the directionality of this relation was not clear; nor did any single executive function emerge as being more involved than others in this relation. Despite this, there was evidence of a reciprocal influence between executive functions and overweight/obesity.

**Conclusions:** This systematic review underlines the presence of a relationship between executive functions and overweight/obesity. Moreover, it seems to suggest a bidirectional trend in this relationship that could be the cause of the failure of interventions for weight reduction. The results of this review highlight the importance of a theoretical model able to consider all the main variables of interest, with the aim to structuring integrated approaches to solve the overweight/obesity problems.

## Introduction

### Rationale

Obesity and overweight, defined as the accumulation of excessive body fat, are risk factors for many chronic diseases, such as hypertension (Jiang et al., 2016) and diabetes (Hauner, 2017) as well as musculoskeletal (McPhail et al., 2014) and respiratory problems (Littleton, 2012). Prospective studies have shown an association between obesity in adulthood and cognitive impairment in old age (Sanderlin et al., 2017). Moreover, obesity appears to be connected to psychopathologies—such as anxiety disorders and depression (Carpiniello et al., 2009; De Wit et al., 2010; Gariepy et al., 2010; Luppino et al., 2010; Carey et al., 2014)—and to social difficulties—such as bullying and social isolation (Kolotkin et al., 2001). The most common risk factors associated with an increase in body weight are poor eating habits and a lack of adequate physical activity (World Health Organization, 2000; Prentice, 2001; Dubbert et al., 2002), which results in a chronic imbalance between individual's needs and energy acquisition (Yumuk et al., 2015).

Conventionally, overweight classifications are made according to the body mass index of an individual (BMI; World Health Organization, 2000). BMI takes into account the weight and height of a person, providing a quantifiable index as the measure of body mass. The WHO considers different severity of overweight: pre-clinical obesity (BMI between 25 and 29.9), obesity class I (BMI between 30 and 34.9), obesity class II (BMI between 35 and 39.9), obesity class III (BMI equal to or higher than 40). An excessive increase in BMI can lead to a higher risk of premature death and a lower quality of life (World Health Organization, 2000). However, some authors focused on other indices that appear to be more sensitive for investigating the relationship between different degrees of overweight and their effects on health, such as waist circumference, waist-to-height ratio, and the body adiposity index (Janssen et al., 2004; Ashwell et al., 2012; Lam et al., 2015). In general, an increase in body fat, in the absence of metabolic and hormonal pathologies (Bray, 1999), is strongly associated with overeating behaviors and excessive ingestion of high-calorific foods that affect the individual's metabolism (McCrory et al., 1999; Ouwens et al., 2003).

The prevalence of obesity or overweight has increased in recent years. In 2014, more than 1.9 billion adults were overweight (World Health Organization, 2015); of these, 600 million were classifiable as obese. Regarding younger people, in 2013 it was estimated that about 42 million children and adolescents between the ages of 5–18 years, and about 12.4% of children below the age of 5 years, were overweight or obese (World Health Organization, 2015; Yumuk et al., 2015). It is expected that around 60% of the world's population will reach critical BMI values by 2030 (Kelly et al., 2008).

Considering these data, it appears useful to investigate the predisposing and exacerbating factors of increases in BMI and body fat, related to overeating behavior. In line with this need, recent years have seen increasing interest in the cognitive mechanisms involved in overweight or obesity (Liang et al., 2014; Forcano et al., 2018). Furthermore, recent studies (Yang et al., 2018) have focused on the relationship between executive functions and obesity to investigate the existence and nature of this association.

### Executive Functions

Executive functions (EFs) is an “umbrella term” (Damasio, 1995; Elliott, 2003; Chan et al., 2008; Diamond, 2013) that includes both complex cognitive processes—such as the resolution of new tasks, the modification of existing behaviors, the planning of new strategies for problem solving, the sequencing of complex actions (Funahashi, 2001; Elliott, 2003), the inhibition of motor or cognitive automatic responses and the control of conflicting information (Diamond, 2013)—and lower-level of cognitive processes, which allow to regulate and control thoughts and actions during goal-directed behavior and involve different cognitive dimensions such as perception and sensation, memory and motivation, attention, reasoning, and problem-solving (Pennington and Ozonoff, 1996).

Although there are various EFs, many studies have centered on three specific processes (Miyake et al., 2000; Diamond, 2013): (i) Cognitive Flexibility (or Shifting), characterized by an attentional shift between tasks or between different mental operations; (ii) Working Memory (or Updating), which includes the updating and monitoring of mental representations in order to respond appropriately to external tasks or stimuli; and (iii) Inhibition, which consists of the voluntary inhibition of dominant or automatic responses for controlling actions, thoughts and emotions, as well as attentional aspects, in order to respond appropriately to the needs of goal-directed behaviors (Miyake et al., 2000; Hofmann et al., 2012; Diamond, 2013). In general, some EFs have been studied more than others due to the presence of cognitive tasks (e.g., Stroop task, Stop Signal Task, Iowa Gambling Task (IGT), Span Task, Maze task) that seem to be more sensitive in the investigation of specific EFs, however there are some limits in their interpretations (Diamond, 2013; Vainik et al., 2013).

Some authors (Grafman and Litvan, 1999; Chan et al., 2008) distinguished between two different groups of EFs: the “cold EFs” and the “hot EFs.” The first—which include verbal reasoning, problem-solving skills, planning, attentional maintenance, cognitive flexibility, response inhibition, and control of conflicting information—are characterized by the absence of emotional processing of stimuli and do not generate emotional arousal (Chan et al., 2008). The second, the hot EFs—which include expectations of punishment-gratification, social behavior, and decision-making—are characterized by the presence of beliefs and desires, and they include a powerful emotional component (Chan et al., 2008). According to Miyake model, these executive domains would be included in cold EFs. However, in daily life, hot and cold EFs work jointly, and both are necessary to direct our behavior. It is essential to underline that EFs are characterized by individual differences, which during life undergo multiple modifications (Jacques and Marcovitch, 2010; Hall and Marteau, 2014), as the reduction of cognitive flexibility and planning with aging (see Jacques and Marcovitch, 2010), or the alterations in inhibition in psychopathology (Nigg, 2000). These differences and changes can also be traced back to the establishment of healthy behaviors, such as eating habits (Hall and Marteau, 2014).

### Executive Functions in Obesity and Overweight

A recent review by Dohle et al. (2018) showed that some studies support the hypothesis that food behaviors affect executive functioning, i.e., healthy eating habits promote the preservation of cognitive functions throughout life (Morris et al., 2005; Smith and Blumenthal, 2016). Other authors are inclined to sustain the opposite point of view, in which cognitive functions are considered as the predictors of food behaviors and, consequently, of body weight changes. According to this view, EFs deficits are considered the cause of inappropriate attitudes to food and represent a trigger for both eating disorders and changes in BMI (Dohle et al., 2018). These different views on the relationship between EFs and eating habits are also observed in the studies that considered the association between obesity/overweight and EFs (Perry, 2004; Pignatti et al., 2006; Davis et al., 2007b; Gonzales et al., 2010).

The theoretical models that consider the relationship between EFs and overweight/obesity are less developed, and usually, they do not focus on specific executive processes. However, it might be interesting to extend this type of studies because they could help in identifying some aspects which are connected to the increase in obesity and related problems. To clarify the nature of the relationship between EFs and overweight/obesity could allow identifying both a causal direction in the relationship between EFs and excessive body weight and the most suitable theoretical model able to explain this relationship.

In general, it could be useful to define whether the studies investigating the relationship between EFs and excessive body weight showed a consensus about the presence of a clear link between the examined variables. In fact, some reviews tried to collect information about this relationship (Fitzpatrick et al., 2013; Vainik et al., 2013; Emery and Levine, 2017; Gettens and Gorin, 2017; Gluck et al., 2017; Yang et al., 2018), but they only confirmed the existence of a correlation between these dimensions, but they were unable to clarify the essence of this relationship, nor the causality. Identifying whether the EFs represent predictors of weight gain (Smith and Robbins, 2013; Chen et al., 2017), or consequences of the increased body weight (Perry, 2004; Sellbom and Gunstad, 2012) still represents an important goal in research.

A review analyzing the relationship between EFs and overweight aimed to examine studies with different experimental designs (cross-sectional, longitudinal) could help in identifying an eventually causal relationship between variables, as well as it could allow understanding how the interactions that emerged in cross-sectional studies, change over time in longitudinal studies. In our view, this represents an essential goal, because it can be useful to both for structuring interventions aimed at reducing risks related to excessive body weight and/or EFs impairment and contributing to the development of a theoretical model. Moreover, studies analyzing the causality between these variables could be a starting point to identify whether some executive domains are more involved than others during body weight gain. An important aspect to consider is the role of every single executive domain in the relationship with overweight/obesity. To identify whether there is a specific EF or some EFs, which influences or are influenced by the excessive body weight could be useful both for the development of a theoretical model on EFs-overweight relationship and for the definition of risk factors related to excessive body weight or impairment in executive domains. Some studies identified impairment in specific EF domains as decision-making, planning and problem solving (for a review see Fitzpatrick et al., 2013), or in inhibition (Gluck et al., 2017). However, generally, the studies identified alteration of EFs, without well-defined the relationship between the single domain of EFs and excessive body weight. This tendency could be due to the different cognitive tasks used and the high number of methodological designs considered in the studies (Vainik et al., 2013).

### Objectives

This systematic review aimed to analyse longitudinal and cross-sectional studies that have investigated the association between EFs and obesity or overweight in the absence of chronic diseases or related eating disorders, trying to add knowledge about the nature of this relationship.

Specifically, the aims of this systematic review are:

- to document the cross-sectional evidence between EFs and overweight/obesity, trying to identify the consensus on the presence of a relationship between EFs and excessive body weight;
- to see if any executive domain has been associated mainly with excessive body weight, considering both positive and negative results;
- to analyse longitudinal studies to assess the causality between EFs and the BMI, considering EFs eventually an outcome or predictor of increase in BMI.

This review represents an attempt to systematize the studies on the relationship between EFs and overweight (Vainik et al., 2013; Emery and Levine, 2017; Yang et al., 2018). The inclusion of longitudinal studies, by also considering different interventions to reduce weight (as in Thiara et al., 2017) could help to clarify the nature of this relationship better. The final aim of this review is to understand how approaching the problems related to excess body weight.

## Method

This systematic review was conducted according to the PRISMA-statement (Liberati et al., 2009; Moher et al., 2009). Online registration of the protocol has not been provided.

### Research Strategies

The systematic review was conducted using PubMed, PsycINFO, PsycArticles, MedLine databases. The following keywords were used: “Executive Function,” “Inhibition,” “Cognitive Inhibition,” “Selective Attention,” “Updating,” “Working Memory,” “Shifting,” Cognitive Flexibility,” “BMI,” “Overweight,” “Obesity,” “Overeating,” “Diet.”

The scripts used for the search are presented in Table 1.

**Table 1:** Script for the systematic research.

	**Script**
Executive function and Obesity	(“executive function”[MeSH Terms] OR (“executive”[All Fields] AND “function”[All Fields]) OR “executive function”[All Fields]) AND (BMI[All Fields] OR (“overweight”[MeSH Terms] OR “overweight”[All Fields]) OR (“obesity”[MeSH Terms] OR “obesity”[All Fields]) OR (“hyperphagia”[MeSH Terms] OR “hyperphagia”[All Fields] OR “overeating”[All Fields]) OR (“diet”[MeSH Terms] OR “diet”[All Fields]))
Inhibition and Obesity	((“inhibition (psychology)”[MeSH Terms] OR (“inhibition”[All Fields] AND “(psychology)”[All Fields]) OR “inhibition (psychology)”[All Fields] OR “inhibition”[All Fields]) OR ((“Cogn Int Conf Adv Cogn Technol Appl”[Journal] OR “cognitive”[All Fields]) AND (“inhibition (psychology)”[MeSH Terms] OR (“inhibition”[All Fields] AND “(psychology)”[All Fields]) OR “inhibition (psychology)”[All Fields] OR “inhibition”[All Fields])) OR (Selective[All Fields] AND (“attention”[MeSH Terms] OR “attention”[All Fields]))) AND (BMI[All Fields] OR (“overweight”[MeSH Terms] OR “overweight”[All Fields]) OR (“obesity”[MeSH Terms] OR “obesity”[All Fields]) OR (“hyperphagia”[MeSH Terms] OR “hyperphagia”[All Fields] OR “overeating”[All Fields]) OR (“diet”[MeSH Terms] OR “diet”[All Fields]))
Working Memory and Obesity	(Updating[All Fields] OR (“memory, short-term”[MeSH Terms] OR (“memory”[All Fields] AND “short-term”[All Fields]) OR “short-term memory”[All Fields] OR (“working”[All Fields] AND “memory”[All Fields]) OR “working memory”[All Fields])) AND (BMI[All Fields] OR (“overweight”[MeSH Terms] OR “overweight”[All Fields]) OR (“obesity”[MeSH Terms] OR “obesity”[All Fields]) OR (“hyperphagia”[MeSH Terms] OR “hyperphagia”[All Fields] OR “overeating”[All Fields]) OR (“diet”[MeSH Terms] OR “diet”[All Fields]))
Cognitive Flexibility and Obesity	(Shifting[All Fields] OR ((“Cogn Int Conf Adv Cogn Technol Appl”[Journal] OR “cognitive”[All Fields]) AND (“pliability”[MeSH Terms] OR “pliability”[All Fields] OR “flexibility”[All Fields]))) AND (BMI[All Fields] OR (“overweight”[MeSH Terms] OR “overweight”[All Fields]) OR (“obesity”[MeSH Terms] OR “obesity”[All Fields]) OR (“hyperphagia”[MeSH Terms] OR “hyperphagia”[All Fields] OR “overeating”[All Fields]) OR (“diet”[MeSH Terms] OR “diet”[All Fields]))

The starting date of the work was January 8th, 2018. All original, “full-text” papers published in international, peer-reviewed journals up to June 10th, 2018 were considered.

### Eligibility Criteria

Selections were made independently by two researchers (FF; MC) and any disagreements resolved by a supervisor (GF). All the studies investigated the relationship between EFs and excessive body weight. Studies including at least one group with overweight or obesity, classified through the international criteria as BMI (World Health Organization, 2000) and BMI percentiles (Flegal et al., 2002), and investigating at least one EF were included. Furthermore, both cross-sectional and longitudinal studies were considered and analyzed separately. For the selection of the articles the following inclusion criteria were used: (a) academic articles published in international, “peer-reviewed” journals; (b) studies written in English; (c) studies on humans with overweight or obesity (BMI higher than 25) at various levels of severity; (d) studies using cognitive tasks to assess EFs; (e) studies including participants aged between 5 and 70 years; (f) cross-sectional and longitudinal studies; (g) studies including different interventions to reduce body weight (bariatric surgery, cognitive remediation therapy, weight-loss programmes that included diets, or physical activity); (h) studies including other psychological variables related to EFs and body weight.

The following exclusion criteria were applied: (a) short report: these type of articles were excluded because after a preliminary analysis of them it was observed that the information reported was too general; (b) studies examining participants with binge eating disorder or other eating disorders; (c) studies focusing on cognitive functions other than executive ones; (d) studies analyzing EFs through self-report questionnaires; (f) studies on obesity of metabolic origin or caused by other medical diseases; (g) studies considering overweight in psychopathological or psychiatric conditions (e.g., depression, schizophrenia, ADHD, etc.). Moreover, for cross-sectional studies, the absence of a normal-weight control group for comparison of executive functioning was an additional exclusion criterion. For the longitudinal researches, both observational and experimental studies were included.

Additionally, in both cross-sectional and longitudinal studies, differences between groups (with normal-weight and overweight), or between different assessment times (pre, post, follow-up) were mainly commented, although regression analyses (continuous BMI) were also considered. Correlational studies were excluded if the method did not include the presence of different conditions of BMI (including both normal-weight and overweight individuals).

### Data Collection Process

According to PICOS (Liberati et al., 2009), the authors extracted from the selected articles information about participants in both the control group and the groups with overweight/obesity (age, BMI, gender), methods (executive tasks used), and main results observed in the EFs tasks.

According to the aims of this review, for the cross-sectional studies, all the results concerning comparisons between overweight/obese groups and normal-weight groups on a cognitive task that assessed one or more EFs were analyzed. For the longitudinal studies, all the results concerning the analysis over time of participants with excessive body weight were examined, including also changes in weight, and executive functioning following either weight-loss programmes or cognitive training. The characteristics of the studies are shown in Tables 3, 4.

### Quality Assessment

A quality assessment analyzed the eligibility of each article by detecting the quality of the studies. This process was aimed to reduce the risk of bias selection and was conducted using a six-point checklist created explicitly for the screening of the studies of this review. For each point, a maximum score of two (high-quality) could be awarded per article: a score of zero corresponded to a low-quality index, a score of 1 to a medium-quality index and a score of 2 to a high-quality index. To derive an overall quality of score of the study, the mean score of each study was multiplied by 100. Studies with a score <75% were considered with high quality, in line with other qualitative analyses (e.g., Varkevisser et al., 2019). The systematic review excluded studies with very low quality (lower than 50%). Table 2 shows the six-point quality assessment checklist. Tables 3, 4 reported the quality assessment for each selected article.

**Table 2:** Checklist for quality assessment.

1) The use of standardized executive tasks^*^.	0 = No standardized tasks; 1 = Use of some non-standardized tasks; 2 = Use of all standardized tasks.
2) Controlling of psychological (e.g. depression, anxiety, emotional dysregulation) and/or physiological variables (e.g. blood values, hormonal and inflammatory aspects).	0 = No control of variables; 1 = Control of psychological or physiological variables; 2 = Control of both psychological and physiological variables.
3) The use of international guidelines for BMI classification.	0 = No international guidelines; 1 = Shared guidelines (i.e. CDC); 2 = International guidelines.
4) Quality of the method description (about executive variables).	0 = Procedures and assessment tools are not well indicated; 1 = Procedures and assessment tools are partially described; 2 = Procedure and assessment tools are well described.
5) Quality of results description (about executive variables).	0 = Executive functioning is not included in the results; 1 = Executive functioning is partially included in the results; 2 = Executive functioning is included in the results.
6) Quality of discussion and conclusion (about executive variables).	0 = Executive functioning is not included in either discussion or conclusion; 1 = Executive functioning is not well included in discussion and conclusion; 2 = Executive functioning is included in both discussion and conclusion.

* *Behavioral tasks widely used in literature for the analysis of a specific executive function*.

**Table 3:** Cross-sectional studies.

	**Participants**					**Method**			**EF Domain**		
**Study**	**Group**	***N***	**Age M (SD)**	**Sex (% female)**	**BMI M (SD)**	**Cognitive task**	**Global EF/other EFs**	**Inhibition**	**Updating**	**Cognitive flexibility**	**Quality of the study**
**STUDY ON ADULT POPULATION**											
Ariza et al. (2012)	OB^1^ NW^2^	42 42	31.81 (6.51) 29.67 (6.97)	67 69	38.3 (7.59) 22.07 (1.97)	TMT^3^ SCWT^4^ WCST^5^ Letter–Number Sequence	–	OB equal to NW	OB equal to NW	OB equal to NW	83.3%
Bongers et al. (2015)	OB NW	185 134	35.19 (7.59) 33.04 (8.15)	71 74	38.18 (6.17) 22.35 (1.63)	Stop–Signal Task Delay Discounting Task (food cue)	OB equal to NW	OB equal to NW	–	–	75.0%
Brogan et al. (2011)	OB NW	42 50	52.24 (10.89) 47.34 (16.34)	71 66	41.45 (9.17) 24.36 (3.78)	IGT^6^	OB poor than NW	–	–	–	83.3%
Catoira et al. (2016)	OB NW	81 32	30 26.5	100 100	35.81 22.56	WCST TMT SCWT Verbal Fluency	–	OB poor than NW	–	OB equal to NW	91.7%
Cohen et al. (2011)	OW NW	42 107	58.9 (8.3) 61.2 (8.0)	48 52	31.8 (6.8) 24.1 (1.4)	SCWT WCST TMT Digit Span	–	OB poor than NW	OB poor than NW	OB poor than NW	66.7%
Danner et al. (2012)	OB OB–BED^7^ NW	18 19 30	44.56 (13.36) 38.05 (10.97) 36.13 (14.09)	100 100 100	30.84 (3) 28.74 (6.25) 22.32 (1.96)	IGT	OB poor than NW	–	–	–	83.3%
Dassen et al. (2018a)	OB NW	82 71	41.12 (12.62) 43.40 (13.44)	64.4 77.5	38.94 (5.24) 22.63 (1.53)	2–Back Task Stop–Signal Task TMT	–	OB poor than NW	OB poor than NW	OB equal to NW	66.7%
Deckers et al. (2017)	OB NW	545 1262	58 (15) 48.9 (16.2)	58 46	31.2 (3.9) 24.9 (2.5)	Concept Shifting Test	–	–	–	OB poor than NW	83.3%
Demos et al. (2017)	OB NW	37 30	46.95 (7.9) 43.97 (8.9)	100 100	33.5 (3.9) 22.7 (1.8)	Food Choice Decision Making Task	OB poor than NW	–	–	–	83.3%
Fagundo et al. (2012)	OB AN^8^ NW	52 35 137	40.5 (11.1) 28.1 (8.2) 24.8 (7)	100 100 100	39.8 (7.4) 17.2 (1.4) 21.5 (2.7)	WCST SCWT IGT	OB poor than NW	OB poor than NW	–	OB poor than NW	75.0%
Frank et al. (2014)	OB ExOB^9^ NW	11 9 11	42.6 (4) 42 (2.8) 36.6 (3.8)	100 100 100	40.2 (0.8) 27.1 (0.9) 21.4 (0.5)	Working Memory Task (food cue)	–	–	OB equal to NW	–	50.0%
Galioto et al. (2013)	OB OW^10^ NW	81 210 288	51.78 (16.96) 50 (17.24) 44.72 (18.37)	55.9 37.5 58	34.67 (5.59) 27.12 (1.45) 22.35 (1.73)	Digit Span Maze Test Switching of Attention Task	OB poor than NW	–	OB poor than NW	OB poor than NW	83.3%
Gameiro et al. (2017)	OB NW	76 38	43.24 (9.05) 40.53 (10.75)	68 71	>30 <25	WCST Go/No–Go Task Color Trait Test Verbal Fluency Motor Series	–	OB poor than NW	–	OB poor than NW	75.0%
Gonzales et al. (2010)	OB OW NW	12 11 9	48.5 (8.6) 52 (5.1) 51.8 (4.3)	50 45 77	34.4 (3.5) 27.4 (1.4) 22.4 (2.2)	Digit Span COWAT^11^ TMT n–Back Task	–	OB equal to OW equal to NW	OB equal to OW equal to NW	OB equal to OW equal to NW	100.0%
Gunstad et al. (2007) [1]	OW NW	140 178	32.40 (9.10) 31.56 (8.71)	46.4 55.1	28.4 (4.42) 22.09 (1.71)	Verbal Interference Task Switching of Attention Task Maze Test	OB poor than NW	OB poor than NW	–	OB poor than NW	75.0%
Gunstad et al. (2007) [2]	OW NW	58 32	60.4 (7.62) 58.34 (6.62)	55.1 53.4	29.17 (3.54) 23.09 (1.59)	Verbal Interference Task Switching of Attention Task Maze Task	OB poor than NW	OB poor than NW	–	OB poor than NW	75.0%
Hendrick et al. (2012)	OB OW NW	13 12 18	34.8 (9.6) 33.2 (16.7) 26.2 (6.7)	100 100 100	33.2 (2.6) 25.6 (2) 20.2 (1)	Stop–Signal Task	–	OB equal to OW equal to NW	–	–	83.3%
Lasselin et al. (2016)	OB–LowCR^12^ OB–HighCR^13^ NW	29 37 20	39.4 (10.5) 37.9 (9) 38.9 (10.1)	62 89 90	40.7 (3.7) 42 (3.8) 22 (3)	IED^14^	–	–	–	OB–HighCR poor thanOB–LowCR; NW	83.3%
Loeber et al. (2012)	OB NW	20 20	47.9 (12.5) 44.9 (11.7)	65 60	38.8 (6.3) 22.6 (1.1)	Go/No–Go Task [food cue] Dot Probe Task (food cue)	–	OB equal to NW	–	–	75.0%
Mole et al. (2015)	OB NW	30 30	44.06 (9.7) 43.59 (10.01)	37 37	32,72 (3.41) 24.11 (2.89)	Delay Discounting Task Stop–Signal Task Information Sampling Task	OB poor than NW	OB equal to NW	–	–	83.3%
Navas et al. (2016)	OB OW NW	20 21 38	32.15 (5.96) 35 (6.31) 33.18 (6.59)	55 52 58	35.5 (2.6) 27.34 (1.59) 22.21 (1.70)	The Ehel of Fortune Task IGT	OB poor than OW; NW	–	–	–	83.3%
Perpiñá et al. (2017)	OB NW	27 39	47.78 (11.46) 31.9 (13.54)	85.2 76.9	43.92 (10.04) 23.21 (3.48)	WCST IGT	OB poor than NW	–	–	OB poor than NW	75.0%
Pignatti et al. (2006)	OB NW	34 20	43.40 (8.13) 46.65 (16.33)	42 50	42.17 (6) 22.16 (1.83)	IGT	OB poor than NW	–	–	–	66.7%
Restivo et al. (2017)	OB–Bar^15^ OB–BarDDM^16^ NW	25 21 20	43.9 (10.7) 43.2 (10.9) 43.8 (11)	92 90 90	44.7 (2.9) 43.7 (4.8) 22.4 (2)	COWAT SCWT WCST Color Trail Test PASAT^17^	–	OB–Bar; OB–BarDDM poor than NW	OB–Bar; OB–BarDDM poor than NW	OB–Bar; OB–BarDDM poor than NW	100.0%
Schiff et al. (2016)	OB NW	23 23	36.2 (9.5) 33.8 (8.9)	78 78	36.2 (5.7) 22.4 (2.2)	Temporal Discounting Task TMT FAB^18^ Simple RT Task Choice RT Task Sterburg Task Simon Task	OB equal to NW	OB equal to NW	OB equal to NW	OB equal to NW	83.3%
Spitoni et al. (2017)	OB NW	24 37	49.8 (13.66) 35.7 (11.2)	79 65	41.1 (8.03) 22.5 (3.01)	BADS^19^-Rule shift Cards Hayling Sentence Completion Task	–	OB poor than NW	–	–	91.7%
Stanek et al. (2013)	OB NW	152 580	43.45 (11.28) 47,66 (18)	84 55	45.23 (6.91) 25.84 (4.97)	Digit Span Switching of Attention Task Verbal Interferences Maze Test	OB poor than NW	OB poor than NW	OB poor than NW	OB poor than NW	83.3%
Stingl et al. (2012)	OB NW	34 34	36.5 (9.5) 38.4 (11)	70 70	30.4 (3.2) 22 (2.1)	N–Back Visual Task (food cue)	–	–	OB poor than NW	–	75.0%
Van der Oord et al. (2018)	OB NW	39 25	42.82 (13.23) 44.9 (15.32)	82.1 72	39.7 (5.31) 22.94 (1.43)	Stop–Signal Task IGT Chessboard Working Memory Task	OB equal to NW	OW equal to NW	OW equal to NW	–	75.0%
Voon et al. (2014)	OB NW	30 30	42.97 (8.59) 43.59 (10.01)	–	32.72 (3.41) 24.11 (2.89)	Premature Responding Task	–	OB equal to NW	–	–	58,3%
**STUDY ON ADOLESCENTS**											
Alarcón et al. (2016)	OB OW NW	18 46 88	14.4 (0.4) 13.8 (0.2) 14.2 (0.1)	33 46 45	%Score 96.9 (0.3) 90 (0.4) 58.9 (1.8)	WS–WM^20^	–	–	OB poor than OW; NW	–	75.0%
Bauer and Manning (2016)	OW NW	74 84	15.59 (1.30) 15.57 (1.24)	100 100	%Score >85° <85°	Visual Working Memory Task	–	–	OW poor than NW	–	75.0%
Calvo et al. (2014)	OB NW	30 32	21.21 (2.45) 21.06 (2.32)	60 53.1	36.36 (6.17) 21.66 (1.78)	Go/No–Go Task Running Memory Continuous Performance Task Standard Continuous Performance Task	–	OB poor than NW	OB poor than NW	–	75.0%
Delgado-Rico et al. (2012)	OW NW	42 21	14.19 (1.38) 14.14 (1.46)	67 48	29.15 (4.51) 19.84 (2.64)	SCWT (Stroop–Switching Performance)	–	OW equal to NW	–	OW equal to NW	66.7%
Fields et al. (2013)	OB OW NW	21 20 20	14.86 (0.85) 15.2 (0.67) 15 (0.86)	52 55 60	>95° 85°- 95° 5°- 85°	Delay Discounting Task Go/No–Go Task Conner's Continuous Performance Test	OB; OW poor than NW	OB equal to OW equal to NW	–	–	83.3%
Galioto Wiedemann et al. (2014)	OB NW	36 36	21.2 (2.9) 20.7 (2)	61.1 50	36.4 (5.7) 22 (1.7)	Go/No–Go Task Running Memory Continuous Performance Task	–	OB poor than NW	OB poor than NW	–	75.0%
Kittel et al. (2017)	OB OB–Bed NW	22 22 22	14.82 (2.63) 14.91 (2.22) 15.23 (2.39)	82 82 82	%score 98.91 (2.3) 99.16 (0.57) 58.91 (24.03)	IGT SCWT	OB equal to OB–Bed equal to NW	OB; OB–Bed poor than NW	–	OB equal to OB–Bed equal to NW	75.0%
Maayan et al. (2011)	OB NW	54 37	17.5 (1.59) 17.32 (1.59)	63.6 56.8	39.86 (9.46) 21.67 (2.49)	SCWT TMT COWAT WRAML–WM^21^	–	OB poor than NW	OB poor than NW	OB poor than NW	91.7%
Moreno-López et al. (2012)	OW NW	36 16	14.22 (1.4) 14.13 (.136)	72 56	28.53 (4.97) 20.26 (2.8)	SCWT	–	OB equal to NW	–	–	75.0%
Nederkoorn et al. (2006)	OB–Bed OB–NBed NW	15 15 31	13.7 13.9 13.7	67 60 61	33 (4.3) 33.5 (4.4) 19.3 (2.0)	Stop–Signal Task Door Opening Task	OB poor than NW	OB poor than NW	–	–	66.7%
Qavam et al. (2015)	OB OW NW	40 40 40	[15–18]	0 0 0	%Score >95° 85°-95° 5°-85°	TOL^22^	OB poor than OW; NW. OW poor than NW	–	–	–	66.7%
Sellaro and Colzato (2017) [1]	OW NW	17 22	23.4 (0.8) 21.2 (0.6)	75 77	27.7 (0.6) 21.9 (0.4)	Stop–Signal Task	–	OW poor than NW	–	–	91.7%
Sellaro and Colzato (2017) [2]	OW NW	19 24	22.9 (1) 20.5 (0.5)	58 79	28.7 (0.6) 21.7 (0.4)	Simon Task	–	OW poor than NW	–	–	91.7%
Steenbergen and Colzato (2017)	OW NW	26 26	20.27 (0.44) 20.36 (0.41)	73 81	27.58 (0.41) 21.67 (0.25)	Switching of Attention Task	–	–	–	OW poor than NW	83.3%
Sweat et al. (2017)	OB NW	108 54	19.6 (1.54) 19.39 (1.52)	63 53.7	35.57 (4.97) 21.45 (1.87)	SWCT TMT TOL	–	OB equal to NW	–	OB equal to NW	83.3%
Vantieghem et al. (2018)	OB NW	62 30	15.8 (1.8) 16 (1.1)	71 47	39.9 (8.19) 20.95 (2.11)	SCWT	–	OB poor than NW	–	–	83.3%
Verbeken et al. (2014)	OW NW	64 66	13.59 (1.62) 12.42 (1.16)	54.2	Adjusted BMI (%) 145.37 (16.27) 102.56 (8.99)	HDT^23^	OW poor than NW	–	–	–	58,3%
Verdejo-García et al. (2010)	OW NW	27 34	14.3 (1.2) 15.29 (0.91)	41 38	31.58 (7.08) 21.01 (1.97)	SCWT Five–Digit Test TMT IGT	OW poor than NW	OW poor than NW	–	OW poor than NW	83.3%
Weller et al. (2008) [1]	OB NW	29 26	19.6 (2.9) 20 (2.6)	100 100	38.4 (6.6) 21.9 (2.3)	Delay Discounting Task	OB poor than NW	–	–	–	75.0%
Weller et al. (2008) [2]	OB NW	19 21	19.2 (1.3) 19.4 (1.5)	0 0	35.4 (4.8) 22.3 (1.2)	Delay Discounting Task	OB equal to NW	–	–	–	75.0%
Wu et al. (2016)	OB NW	19 20	21.3 (2.6)	74 70	33 (2.9) 22.2 (2.2)	SCWT TMT Verbal Fluency Digit Span	–	OB equal to NW	OB poor than NW	OB poor than NW	58.3%
Yau et al. (2014)	OB NW	30 30	17.64 (1.62) 17.22 (1.55)	57 63	35.47 (5.88) 21.12 (2.18)	TMT WCST SCWT COWAT	–	OB equal to NW	OB poor than NW	OB poor than NW	91.7%
**STUDY ON YOUNG ADULTS**											
Coppin et al. (2014)	OB OW NW	17 16 16	25.17 (4.39) 24.94 (4.55) 24.25 (4.25)	53 44 56	36.02 (6.54) 27.63 (1.49) 22.43 (1.45)	CCPT^24^	–	–	OB, OW poor than NW	–	91.7%
Yadava and Sharma (2014)	UW NW NW2 OW OB	39 50 58 58 25	26.9 [20–42]	100	<18.5 18.5–22.9 23–24.9 25–29.9 30	Digit Symbol Test SCWT Ascending Digit Task	–	OB poor than NW	OB poor than NW	OB poor than NW	75.0%
**STUDY ON CHILDS**											
Blanco-Gómez et al. (2015)	OB OW NW	39 149 316	[6–10]	49 53 50	%Score >97 95–97 <95	Children's Color Traits Test (1,2) Five Digit Test	–	OB poor than OW; NW	–	OB poor than OW; NW	83.3%
Bozkurt et al. (2017)	OB NW	92 55	11.85 (2.43) 11.9 (2.96)	56 54	29.73 (2.33) 21.07 (1.81)	FTT^25^ SDC^26^ SCWT SAT^27^ CPT^28^	OB poor than NW	OB poor than NW	OB poor than NW	OB poor than NW	83.3%
Gentier et al. (2013)	OB NW	19 19	9.8 (1.5) 9.9 (1.5)	47 47	Cut–off (Cole et al. 2000) 21.62 (3.51) 16.48 (1.76)	Four Choice Reaction Time Task	OB poor than NW	–	–	–	58.3%
Goldschmidt et al. (2018)	OW–LC^29^ OW–C^30^ NW–C^31^	26 34 15	10.2 (0.9) 10.8 (1.1) 10.4 (1.1)	61 56 60	z–score 2.08 (0.47) 2.02 (0.47)	Flanker Task DCCST^32^ IGT TOL List Sorting	OB–LC; OB–C poor than NW–C	OB–LC equal to OB–C equal to NW–C	OB–LC; OB–C poor than NW–C	–	75.0%
Kamijo et al. (2012a)	OB OW NW	30 26 70	9 (0.5) 8.7 (0.6) 8.9 (0.6)	100 100 100	%score >95°>85° >5°	Go/No–Go Task	–	OB poor than NW	–	–	75.0%
Kamijo et al. (2012b)	OB NW	37 37	9 (0.5) 8.9 (0.5)	51 51	%score >95° 5°-85°	Go/No–Go Task	–	OB poor than NW	–	–	75.0%
Kamijo et al. (2012c)	OB NW	37 37	8.9 (0.6) 8.8 (0.6)	54 54	%score 98 (1.4) 56.8 (19.9)	Flanker Task	–	OB poor than NW	–	–	75.0%
Pearce et al. (2018) [1]	OB NW	41 37	13.3 (3.4) 13.1 (2.7)	54 30	%Score 98.8 (1.2) 58.3 (26.1)	BART^33^	OB equal to NW	–	–	–	75.0%
Pearce et al. (2018) [2]	OB NW	29 30	11.4 (2.6) 11.9 (2.6)	48 40	%Score 98.5 (1.3) 60.7 (25.4)	Stop–Signal Task N–Back Task	–	OB equal to NW	OB equal to NW	–	75.0%
Reyes et al. (2015)	OW NW	93 92	10.2 (1) 10.3 (0.2)	44 46	z–score 1.9 (0.6) 0.1 (0.5)	SCWT Go/No–Go Task	–	WB poor than NW		–	58.3%
Skoranski et al. (2013)	OB NW	28 32	12.8 (2.4) 12.8 (2.5)	79 47	%Score >85° 5°- 85°	Arrow Task	–	OB poor than NW	–	–	58.3%
Tsai et al. (2016)	OB NW	26 26	(month) 114.58 (3.69) 113.73 (3.85)	31 31	%Score >95° 5°-85°	Posner Paradigm Task	–	OB poor than NW	–	–	58.3%
Wu et al. (2017)	OB NW OW	44 23 92	12.38 (1.22) 11.78 (1) 11.93 (0.92)	32 26 56	>30 25–30 <25	Digit Span Memory Task (digits; digit–food cue; digit–cartoon)	–	–	OW poor than NW	–	66.7%

*1OB, Obese; 2NW, Normal-Weight; 3TMT, Trail Making Test; 4SCWT, Stroop Color-Word Task; 5WCST, Wisconsin Card Sorting Test; 6 IGT, Iowa Gambling Task; 7OB-BED, Obese with Binge Eating Disorder; 8AN, Anorexia Nervosa; 9ExOB, Normal-weight people who were previously obese; 10OW, Overweight; 11COWAT, Controlled Oral Word Association Task; 12OB-LowCR, Obese with low sensitivity to C-reactive protein; 13OB-HighCR, Obese with high sensitivity to C-reactive protein; 14IED, Intra/Extra-dimensional set shift test; 15OB-Bar, Obese and on the waiting list for bariatric intervention; 16OB-BarDDM, Obese and on the waiting list for bariatric intervention with Major Depressive Disorder; 17PASAT, Paced Auditory Serial Attention Test; 18FAB, Frontal Assessment Battery; 19 BADS, Behavioral Assessment of the Dysexecutive Syndrome; 20WS-WM, Working Memory Task of Wechsler Scale; 21WRAML-WM, Wide-Range Assessment of Memory and Learning-Working Memory; 22TOL, Tower of London; 23HDT, Hungry Donkey Task; 24CCPT, Conditioned Cue Preference Test; 25FTT, Finger-Tapping Test; 26SDC, Symbol Digit Coding; 27SAT, Shifting Attention Test; 28CPT, Continuous Performance Test; 29OW-LC, Overweight with high loss of control; 30OW-C, Overweight with low loss of control; 31NW-C, Normal-weight with low loss of control; 32DCCST, Dimensional Change Card Sort task; 33BART, Balloon Analog Risk Task*.

**Table 4:** Longitudinal Studies.

	**Participants**					**Method**			**EF Domain**				
**Study**	**Group**	***N***	**Age M (SD)**	**Sex (% female)^a^**	**BMI**^b^*M*(*SD*)****	**Treatment**	**Cognitive task**	**Program/follow–up**	**Global EF/other EFs**	**Inhibition**	**Updating**	**Cognitive flexibility**	**Quality of the study**
^*^Allom et al. (2018)	OB–CRT^1^ OB–C^2^	42 38	41.39 (7.85)	86	39.76 (7.53)	Cognitive Remediation Therapy	WCST^3^ TMT^4^	3 months	OB–CRT ↑ OB–C =	–	.	OB–CRT ↑ OB–C =	83.3%
Alosco et al. (2014a)	OB–AD^5^ OB–NAD^6^	14 80	40 (11.42) 45.1 (10.99)	21.4 15	T1.45.17 (5.02) T2.37.85 (5.43) T1.46.07 (5.33) T2.38.06 (4.86)	Bariatric Surgery [Alzheimer History]	TMT Maze Task	12 weeks	OB–AD = OB–NAD =	–	.	OB–AD = OB–NAD =	75.0%
Alosco et al. (2014b)	OB–Bar^7^ OB–C	63 23	42.29 (11.42) 41.13 (12.55)	90.5 95.7	T1.46.5 (5.26) T2.31.34 (6.42) T1.40.9 (5.24) T2.40.9 (5.64)	Bariatric Surgery	Digit Span Switching Attention Task Maze Task	24 months	OB–Bar ↑ OB–C =	–	OB–Bar ↑ OB–C =	OB–Bar ↑ OB–C =	91.7%
Alosco et al. (2014c)	OB–Bar	78	43.5 (10.59)	82.1	T1.46.63 (5.28) T2.30.51 (5.39)	Bariatric Surgery	Switching of Attention Task Maze Task	12 months	OB–Bar ↑	–	–	OB–Bar ↑	66.7%
Alosco et al. (2014d)	OB–Bar	50	44.08 (10.76)	92	T1.46.61 (5.27) T2.32.35 (6.57) T3.33.02 (6.27)	Bariatric Surgery	Digit Span Switching of Attention Task Verbal Interference Maze Task	I. 36 months II. 48 months (LD)^8^	I.OB–Bar ↑ II. OB–Bar ↑	I.OB–Bar ↑II. OB–Bar ↑	I.OB–Bar ↑ II. OB–Bar ↑	I.OB–Bar ↑II. OB–Bar ↑	83.3%
Alosco et al. (2015)	OB–Bar	84	43.86 (10.39)	83.3	T1.46.88 (6.08) T2.30.05 (5.39)	Bariatric Surgery	Digit Span Switching of Attention Task Verbal Interference Task	12 months	–	OB–Bar ↑	OB–Bar ↑	OB–Bar ↑	83.3%
^*^Augustijn et al. (2018)	OB	T1.32 T2.30	9.6 (1.1)	T1.56 T2.60	Z scores T1. 2.7 (0.3) T2.2.0 (0.4)	Weight Loss Program	CANTAB^9^	6–10 months	OW↑	OW↑	OW↑	OW =	75.0%
Bryan and Tiggemann (2001)	OB–WL^10^ OB–C	42 21	48.9 (8.2) 50.9 (7.3)	100 100	T1.34.1 (4.3) T1.35.2 (4.8)	Weight Loss Program	TMT WCST Self–Ordered Piniting Task Initial Letter Fluency Excluded Letter Fluency Digit Span	12 weeks	–	OB–WL ↑ OB–C =	OB–WL = OB–C =	OB–WL = OB–C =	91.7%
^*^Dassen et al. (2018b)	OW–WMT^11^ OW–C	T1.51 T2.34 T1.40 T2.36	47.97 (10.69)	74.7	T1.30.96 (3.64) T2.29.95 (3.46) T1.30.49 (3.97) T2.30.17 (4.14)	Working Memory Training	2–Back Task	25 session	–	–	OW–WMT↑	–	66.7%
Davis et al. (2007a)	OW–HE^12^ OW–LE^13^ OW–NE^14^	32 33 29	9.2 (0.84)	60	z–score 2.1 (0.4)	Weight Loss Program: Aerobic Exercise	CAS^15^: Planning Subscales for EF	15 weeks	OW–HE↑	–	–	–	66.7%
Davis et al. (2011)	OW–HE OW–LE OW–NE	56 55 60	9.3 (1.0)	56	z–score 2.1 (0.4)	Weight Loss Program: Aerobic exercise	CAS: Planning Subscales for EF	13 weeks	OW–HE↑	–	–	–	75.0%
Deckers et al. (2017)	OB NW	T1.545 T2.190 T1.1262 T2.834	T1.58 (15) T2.48.9 (16.2) T1.48.9 (16.2) T2.46.7 (14.9)	I.58 II.59 I.46 II.43	T1.31.2 (3.9) T2.28.7 (2.4) T1.24.9 (2.5) T2.24.8 (2.4)	–	Concept Shifting Test	6 years12 years	OB = NW =	–	–	OB = NW =	83.3%
Demos et al. (2017)	OB–WL NW	37 30	46.95 (7.9) 43.97 (8.9)	100 100	T1.33.5 (3.9) T1.22.7 (1.8)	Weight Loss Program	Food Choice Decision Making Task	12–16 weeks	OB–WL↑	–	–	–	83.3%
Galioto et al. (2015)	OB–Bar	72	43.55 (10.21)	81.7	T1.46.32 (5.51) T2.30.18 (5.25)	Bariatric Surgery	Digit Span Switching of Attention Task Verbal Interference Verbal Fluency	12 months	OB–Bar↑	OB–Bar↑	OB–Bar↑	OB–Bar↑	91.7%
^*^Galioto et al. (2016)	OB	23	50.35 (15.11)	68	44.21 (8.82)	Weight Loss Program	Dot Counting Task N–Back Task Set Shifting Task Unstructured Task Flanker Task	8 weeks	–	OB↑	OB =	OB↑	100.0%
^*^Kulendran et al. (2014)	OB–WL	53	14.28 (1.15)	60	T1.33.75 (7.9)	Weight Loss Program	Stop–Signal Task Delay Discounting Task	2–8 weeks	–	OB–WL↑	–	–	83.3%
^*^ Kulendran et al. (2017)	OB–Bar	45	43.42 (13.06)	31	T1.44.25 (6.34) T2.35.51 (7.08)	Bariatric Surgery	Stop–Signal Task (food–cue) Temporal Discounting Task	6 months	–	OB–Bar↑	–	–	75.0%
^*^Pauli-Pott et al. (2010)	OW	111	11.1 (2.0)	57	95° percentile	Weight Loss Program	Go/No–Go Task Interference Task	1 year	–	OW↑	–	–	91.7%
Pearce et al. (2017)	OB–Bar OB–C NW	10 14 12	17 (1.37) 16.42 (1.35) 16.51 (1.27)	60 71 50	T1.47.18 (6.98) T1.45.32 (8.19) T1.21.57 (2.59)	Bariatric Surgery	Verbal N–Back Test Ballon analog risk task	4 months	OB–Bar = OB–C = NW =	–	OB–Bar = OB–C = NW =	(DM area shows a reduction of activation in OB–Bar after the surgery)	83.3%
Raman et al. (2018)	OB–CRT OB–C	42 38	40.6 (2.4) 42.2 (8.8)	86	39.2 (7.4) 40.3 (7.8)	Computerized Cognitive Remediation Therapy	WCST TMT	8 weeks 3 months	–	–	–	OB–CRT ↑OB–C =	83.3%
^*^Spitznagel et al. (2013)	OB–Bar	84	44.75 (9.99)	79.8	T1.46.13 (5.80) T2.37.46 (4.99) T3.31.07 (6.44)	Bariatric Surgery	Switching of Attention Task Digit Span Maze Task	I.12 weeks II. 12 months	I.OB–Bar = II.OB–Bar↑	–	I.OB–Bar = II.OB–Bar↑	I.OB–Bar = II.OB–Bar↑	75.0%
^*^Spitznagel et al. (2014)	OB–Bar	55	45 (10.28)	87.3	T1.45.11 (5.11) T2.37.23 (4.76) T3.31.69 (5.84)	Bariatric Surgery	Digit Span Switching of Attention Verbal Interference Verbal Fluency Maze Task	12 weeks 36 months	OB–Bar↑	OB–Bar =	OB–Bar↑	OB–Bar =	83.3%
^*^Stinson et al. (2018)	OW	46	37.2 (10.2)	24	28.3 (6.7)	–	IGT WCST SCWT	32 ± 25 months	OW =	OW**↓**	–	OW =	100.0%
Vantieghem et al. (2018)	OB–WL NW	62 30	15.8 (1.8) 16 (1.1)	71 47	T1.39.9 (8.19) T2.32.21 (7.14) 20.95 (2.11)	Weight Loss Program	SCWT	30 weeks	–	OB–WL↑	–	–	83.3%
Verbeken et al. (2013)	OB–EFT^18^ OB–C	22 22	11.50 (1.60) 11.41 (1.93)	50 41	Adjusted BMI T1. 131.58 (21.70) T1. 132.91 (15.98)	Executive Function Training	Corsi Block–Tapping Task Stop–Signal Task	Post–Test 8 weeks 12 weeks	OB–EFT ↑ OB–C =	–	–	–	58.3%
Witbracht et al. (2012)	OB	29	32.7 (9.2)	100	32 (2.6)	Weight Loss Program	IGT	12 weeks	OB↑	–	–	–	83.3%
Xie et al. (2017)	OB–WL OB–C	30 28	15.07 (0.83) 15.18 (0.39)	27 36	T1.32.83 (3.84) T2.29.19 (3.52) T1.30.90 (1.95) T2.30.47 (2.13)	Weight Loss Program	Flanker Task	4 weeks	–	OB–WL↑OB–C =	–	–	75.0%
^*^Xu et al. (2017)	OB–WL	31	18.2 (3.2)	39	34.4 (4.8)	Weight Loss Program	SCWT	4 weeks program	–	OB–WL ↑	–	–	83.3%

* EF-predicted weight loss.

a Percentage of females.

b Body Mass Index. ↑, Better performance after treatment; ↓, Worse performance after treatment. No differences.

1 OB-CRT, Obese and in Cognitive Remediation Therapy Treatment;

2 OB-C, Obese-Control (No treatment group);

3 WCST, Wisconsin Card Sorting Test;

4 TMT, Trail Making Test;

5 OB-AD, Obese with history of Alzheimer's;

6 OB-NAD, Obese with no history of Alzheimer's;

7 OB-Bar, Obese and subjected to bariatric surgery;

8 LD, Loss Data;

9 CANTAB, Cambridge Neuropsychological Test Automated Battery;

10 OB-WL, Obese and subjected to a weight-loss programme;

11 OB-WMT, Obese and subjected to Working Memory Training;

12 OW-HE, Overweight and subjected to high-exercise training;

13 OW-LW, Overweight and subjected to low-exercise training;

14 OW-NE, Overweight with no exercise training;

15 CAS, Cognitive Assessment System;

16 SCWT, Stroop Color-Word Task;

17 IGT, Iowa Gambling Task (for decision-making);

18 *OB-EFT, Obese and subjected to Executive Function Training*.

## Results

### Study Selection

The initial search produced 1,817 articles. After excluding 614 duplicates, 922 articles were rejected according to an analysis of both title and abstract, leaving a final total of 281 studies to be reviewed and subjected to the quality assessment.

**Figure 1:**
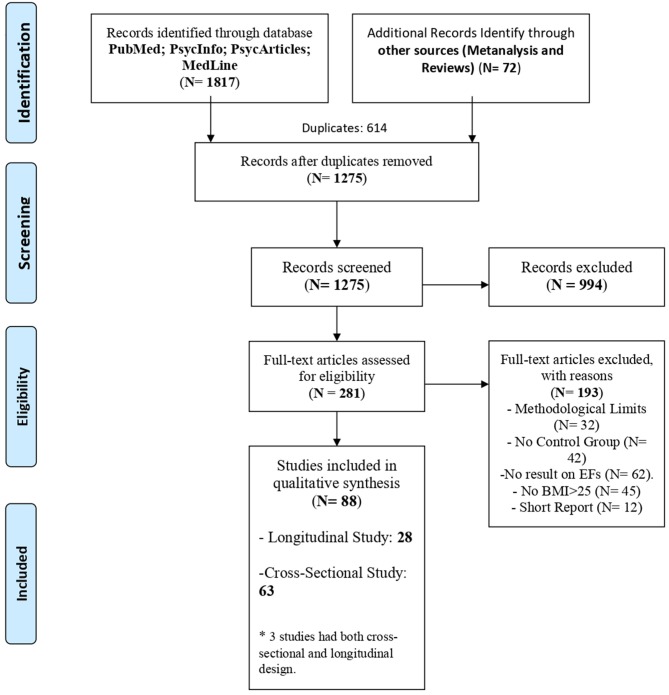
Flow chart.

At the end of the review process, 88 articles remained. The flow chart (Figure 1) shows the study selection process, including the number of studies found, the assessment process and the reasons for the exclusion of the articles.

The 88 selected articles were categorized according to the experimental design. Sixty-three studies used a cross-sectional design, and twenty-eight studies used a longitudinal design (see Tables 3, 4). Three studies (Deckers et al., 2017; Demos et al., 2017; Vantieghem et al., 2018) used both cross-sectional and longitudinal design. These studies considered the differences between participants with normal-weight and participants with overweight or obesity and analyzed the differences in executive performances during the time. For this reason, they were considered in both sections of the review.

### Quality Assessment for Risk Bias

**Figure 2:**
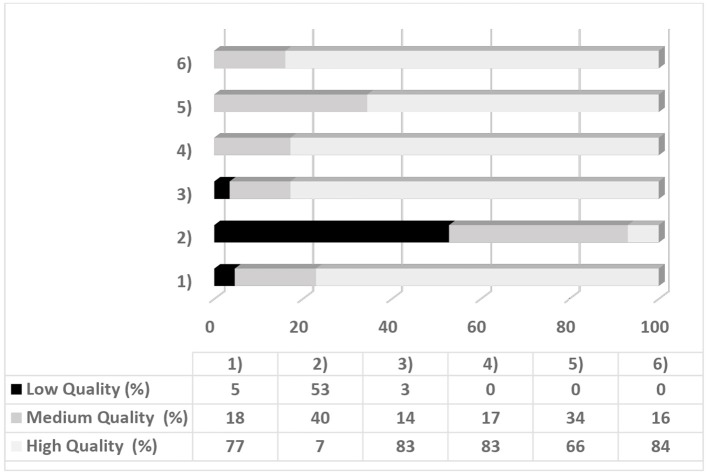
Percentage of the studies and quality levels for each point of tool assessment.

Seventy-nine per cent of the studies (*N* = 70) were of high quality, while 20% (*N* = 18) were of low quality. Figure 2 shows the percentage of studies per quality level for each point on the assessment tool. Overall, studies showed higher quality in their results and discussion sections. Conversely, lower scores were found for the control of psychological and physiological variables (Figure 2). The selection of the articles for the systematic review was justified by the good quality of each study, explicitly considering the results on EFs. In general, despite a large number of the selected studies, the high quality of the studies may have reduced the risks of misinterpretation of the results.

### Cross-Sectional Studies

Systematic searching gave 63 cross-sectional studies that met the inclusion criteria (see Table 3). Of these studies, twenty-nine involved adult participants (aged over 30 years), twenty examined adolescents (aged 12–22 years), two studies looked at young adults (aged 23–30 years) and finally twelve studies investigated the relationship between EFs and excessive body weight in children (aged >12 years) (see Table 3).

Only nine studies had a higher proportion of males than females (Pignatti et al., 2006; Verdejo-García et al., 2010; Gentier et al., 2013; Mole et al., 2015; Qavam et al., 2015; Reyes et al., 2015; Alarcón et al., 2016; Tsai et al., 2016; Wu et al., 2017). Furthermore, Weller et al. (2008) performed two different analyses to examine samples of males and females independently.

All the studies used BMI and the related WHO classification to assign participants to different overweight groups. For children and adolescents, the guidelines for using percentiles recommended by WHO or Center for Disease Control and Prevention (CDC) were employed (Flegal et al., 2002; de Onis et al., 2007), except in two studies (Reyes et al., 2015; Goldschmidt et al., 2018) where z scores for CDC classification were used (Harrington et al., 2013).

Studies focused mainly on differences in executive functioning between individuals with obesity and normal-weight; thirteen analyzed differences between participants with normal-weight and overweight; thirteen studies investigated differences in performance between participants with obesity, overweight, and normal-weight (see Table 3).

Most of the studies reported a significant difference between the groups in executive functioning, confirming the relationship between excessive body weight and executive dysfunctions. Only thirteen studies reported no differences (Gonzales et al., 2010; Ariza et al., 2012; Delgado-Rico et al., 2012; Hendrick et al., 2012; Loeber et al., 2012; Moreno-López et al., 2012; Frank et al., 2014; Voon et al., 2014; Bongers et al., 2015; Reyes et al., 2015; Schiff et al., 2016; Sweat et al., 2017; Van der Oord et al., 2018).

## Executive Functions in Cross-Sectional Studies

### Cognitive Flexibility

The tasks most commonly used to assess cognitive flexibility were the Wisconsin Card Sorting Test (WCST) (Milner, 1963), the Trail Making Test (TMT, AB) (Reitan, 1958) and the Switching of Attention Task (Rogers and Monsell, 1995) (see Table 3).

Twenty-seven studies assessed the differences between groups on cognitive flexibility (see Table 3); only eight of them found no differences in cognitive flexibility between normal-weight and overweight/obese groups (Gonzales et al., 2010; Ariza et al., 2012; Delgado-Rico et al., 2012; Catoira et al., 2016; Schiff et al., 2016; Kittel et al., 2017; Sweat et al., 2017; Dassen et al., 2018a). In general, the results showed greater difficulty in performing tasks involving this function in participants with obesity compared to those with normal-weight. Furthermore, the study by Blanco-Gómez et al. (2015) highlighted a further difference: compared with participants with overweight, participants with obesity showed higher flexibility deficits.

### Inhibition

The most common cognitive tasks used to measure inhibitory control were the Stroop Color-Word Task (Stroop, 1935) and the Stop-Signal Task (Lappin and Eriksen, 1966) (see Table 3).

Forty-five studies investigated the relationship between inhibitory control and excessive body weight (see Table 3). Of these, seventeen studies reported no differences between the groups (Gonzales et al., 2010; Ariza et al., 2012; Delgado-Rico et al., 2012; Hendrick et al., 2012; Loeber et al., 2012; Moreno-López et al., 2012; Stingl et al., 2012; Fields et al., 2013; Voon et al., 2014; Yau et al., 2014; Bongers et al., 2015; Schiff et al., 2016; Wu et al., 2016; Goldschmidt et al., 2018; Pearce et al., 2018; Van der Oord et al., 2018). The remaining studies reported lower inhibitory control in obese than in normal-weight participants.

### Working Memory

The Digit Span Test (in particular the Backwards version) (Reynolds, 1997), and the N-Back Test (Kane et al., 2007) were used in various versions (see Table 3) to investigate differences in working memory performance.

Of the twenty-four studies that analyzed the relation between overweight/obesity and working memory (see Table 2), six observed no differences between groups (Gonzales et al., 2010; Ariza et al., 2012; Frank et al., 2014; Restivo et al., 2017; Pearce et al., 2018; Van der Oord et al., 2018). The remaining studies found that participants with overweight/obesity performed worse than normal-weight participants on working memory tasks; moreover, obese participants performed worse than participants with overweight (Coppin et al., 2014; Alarcón et al., 2016).

### Decision-Making, Planning, and Problem-Solving

The tasks used to assess decision-making, planning and problem-solving were the IGT (Bechara et al., 2005) and the Delay Discounting Task (Richards et al., 1999) (see Table 3).

Twenty-six studies (see Table 3) investigated differences in performances between groups on tasks involving complex EFs such as decision-making, planning and problem-solving. Among these studies, only six (Bongers et al., 2015; Mole et al., 2015; Schiff et al., 2016; Kittel et al., 2017; Pearce et al., 2018; Van der Oord et al., 2018), individuals with obesity performed worse than those with normal-weight on decision making, planning and risk-taking. Furthermore, Schiff et al. (2016), despite of they observed no clear between differences in decision-making, found that the group with obesity responded differently in terms of gratification mechanisms connected with food (as measured by the Temporal Discounting Task), in fact, they showed more sensitivity to reward stimuli than normal-weight group. Weller et al. (2008), using the Delay Discounting task, found that women affected by obesity, compared to women with normal-weight, preferred an immediate reward than a major one after some time. This difference was not observed in men (Weller et al., 2008).

## Discussion

The analysis of the cross-sectional studies confirmed the existence of a relationship between overweight/obesity and EFs, even if it did not indicate the direction of this relationship. Many types of cognitive tasks were used to investigate executive functioning, but, despite this heterogeneity, the results were consistent. However, the very different demands of the tasks used did not allow determining whether one single EF is more closely involved than the others in the relationship with overweight/obesity, though the most analyzed EF related to excessive body weight is Inhibition (see Table 3). The studies that failed to confirm a relationship between EFs and overweight/obesity used a small sample size (Hendrick et al., 2012; Schiff et al., 2016), or a high number of cognitive tasks (Gonzales et al., 2010)

The present systematic review included studies that take into account people with different ages considering from children to the elderly. This choice was aimed to investigate whether the relationship between EFs and overweight/obesity presents similar characteristics, regardless of the age of the participants. The results of the review confirmed the relationship between EFs and overweight both in studies examining adults and young adults (Gunstad et al., 2007; Fagundo et al., 2012; Coppin et al., 2014) and in those that looked at children (Yadava and Sharma, 2014; Bozkurt et al., 2017) and adolescents (Nederkoorn et al., 2006; Galioto Wiedemann et al., 2014). These results prevent us from making inferences about the causality of this relationship over a lifespan but highlight the existence of a negative relationship between executive performances and overweight, regardless of the age considered.

Many studies tried to control for certain variables (gender, age, and education) that might influence executive performance, by matching samples or controlling the effects of these variables through statistical analysis (Gunstad et al., 2007; Deckers et al., 2017; Kittel et al., 2017; Perpiñá et al., 2017). This methodological aspect highlighted the existence of some dimensions (e.g., demographical variables as gender or educational level) that might influence the relation between body weight and EFs; therefore, considering these variables can contribute to further strengthen the results (Kittel et al., 2017).

Generally, the analyzed studies used suitable inclusion criteria that allow excluding individuals with chronic medical conditions, psychological diseases or eating disorders, in order to avoid an effect of these dimensions on the observed results (Fagundo et al., 2012; Galioto et al., 2013; Galioto Wiedemann et al., 2014). Moreover, in some studies, physiological differences between participants with normal-weight and overweight/obesity were reported. In particular, participants with severe obesity showed worse values, in blood pressure, cholesterol levels, insulin resistance (Maayan et al., 2011; Perpiñá et al., 2017) and levels of glycolic metabolism activation although, in the absence of pathological medical conditions in line with well-known results (Heymsfield and Wadden, 2017).

Both psychopathological and physiological aspects related to obesity, and specifically with severe obesity, have an impact on the executive functioning and consequently with the performances in executive tasks; therefore these variables should be controlled in further studies.

Although the cross-sectional studies showed no clear direction in the overweight–executive functioning relationship, many of the authors have advanced various hypotheses (Gonzales et al., 2010; Galioto Wiedemann et al., 2014). For example, Kamijo et al. (2012a) hypothesized that ineffective inhibitory control of the prefrontal cortex would cause excessive consumption of calories that is directly associated with an increase in body fat. Moreover, other authors considered also the dopaminergic mechanism involved in executive processing as related to weight variations (Arnsten and Li, 2005). Neuroimaging studies of individuals with obesity have shown an association between the hypoactivation of dopaminergic D2-receptors and a decrease in neural metabolism in the areas most involved in executive functioning (Volkow et al., 2011). Furthermore, dopamine is also implicated in the reward system (Volkow et al., 2011; Smith and Robbins, 2013). This neural system resulted impaired in individuals with excessive body weight, and alterations of this system could influence the approach to food in terms of favoring the consumption of high-calorie foods to achieve higher gratification (Schiff et al., 2016). All these findings could support theoretical models on the genesis of obesity (Davis et al., 2007b; Smith and Robbins, 2013) that view changes in executive functioning as one of the leading causes of weight gain. The hypothesis of executive dysfunctions as a cause of inappropriate eating behavior could partially support the theoretical model of Food Addiction, in which the excessive consumption of food is characterized by behavioral aspects similar to those defining other substance addiction diseases (Wang et al., 2004; Smith and Robbins, 2013).

Nevertheless, other authors viewed executive deficits as a consequence of obesity, recognizing it as a cause of neurophysiological and metabolic diseases, such as changes in insulin sensitivity (Gonzales et al., 2010), inflammatory processes as a result of body fat accumulation (Lasselin et al., 2016), and changes in cerebrovascular blood flow (Verdejo-García et al., 2010; Qavam et al., 2015). These alterations could be the cause of structural changes (e.g., a reduction of the orbitofrontal cortex) (Cohen et al., 2011) or functional changes (e.g., reduced functional connectivity of executive networks) (Tsai et al., 2016) in the cerebral areas involved in executive functioning. This vision seems to be in line with the Neuroinflammation Model (Perry, 2004) in which high BMI appears to result in systemic inflammation, which negatively affects cognitive functions including executive ones (C-reactive protein and interleukin would play an essential role in this process; Bourassa and Sbarra, 2017), and with the model proposed by Sellbom and Gunstad (2012) in which the changing in blood flow and metabolism of the frontal lobes as well as the atrophy of the frontal and temporal lobes would cause an impairment in inhibitory control resulting in an increase in overeating behaviors (Sellbom and Gunstad, 2012).

The consistent results confirming the relationship between EFs and obesity suggests that even a moderate increase in body weight may be associated with a decrease in executive performances (Verdejo-García et al., 2010; Cohen et al., 2011; Sellaro and Colzato, 2017). These views are supported by results obtained comparing groups of participants with normal-weight, overweight and obesity, in which differences in performances also emerged between overweight and obesity conditions (Galioto Wiedemann et al., 2014; Wu et al., 2017).

Another aspect highlighted by the cross-sectional studies is the role of certain psychological variables related to BMI (Catoira et al., 2016; Restivo et al., 2017) that appear to modulate the relationship between EFs and excessive body weight. Indeed, the presence of high levels of anxiety and depression in individuals with obesity, even in the absence of established psychopathologies, appears to result in worse executive performances (Restivo et al., 2017). These findings could be linked to the theoretical model of Emotionally-Driven Eating (Dallman, 2010), which postulated that overeating, related to overweight, is a dysfunctional attempt to regulate emotions in people characterized by a deficit in emotion regulation.

### Longitudinal Studies

Our systematic search allows selecting twenty-eight longitudinal studies investigating executive functioning in individuals with overweight or obesity (see Table 4). Of these, eighteen examined adult participants (aged more than 30 years), five looked at children (aged <12 years) and five involved adolescents (aged 12–22 years) (see Table 4).

All studies used BMI to classify overweight and obesity, although z-scores (Davis et al., 2007a, 2011; Augustijn et al., 2018), percentiles (Pauli-Pott et al., 2010), or adapted BMI scores (Verbeken et al., 2014) were used in studies involving children.

Five studies (Alosco et al., 2014a; Kulendran et al., 2017; Xie et al., 2017; Xu et al., 2017; Stinson et al., 2018) reported having a significantly higher percentage of males than females in their sample.

Twelve studies (Bryan and Tiggemann, 2001; Davis et al., 2007a, 2011; Pauli-Pott et al., 2010; Witbracht et al., 2012; Kulendran et al., 2014; Galioto et al., 2016; Demos et al., 2017; Xie et al., 2017; Xu et al., 2017; Augustijn et al., 2018; Vantieghem et al., 2018) analyzed the effects of non-invasive programmes aimed at weight-loss on the relationship between BMI and EFs: some interventions integrated various modalities of treatment, specifically diet and physical activity (Pauli-Pott et al., 2010; Kulendran et al., 2014; Galioto et al., 2016; Demos et al., 2017; Xie et al., 2017; Xu et al., 2017; Vantieghem et al., 2018); while others focused only on diet programmes (Bryan and Tiggemann, 2001; Witbracht et al., 2012) or physical activity (Davis et al., 2007a, 2011). Furthermore, two studies (Kulendran et al., 2014; Augustijn et al., 2018) provided residential interventions, with treatment lasting from four (Davis et al., 2011) to fifty-2 weeks (Pauli-Pott et al., 2010). In all the studies, at least two measurements were taken: one before and one after the procedure.

Ten studies examined the effects of bariatric surgery on the executive functioning in participants with severe obesity (Spitznagel et al., 2013, 2014; Alosco et al., 2014a,c,d, 2015; Galioto et al., 2015; Kulendran et al., 2017; Pearce et al., 2017). The analysis of EFs was performed before surgery and at follow-up, with time intervals ranging from 12 weeks (Spitznagel et al., 2013, 2014) to 48 months (Alosco et al., 2014d). In some cases, more than one follow-up was carried out (Spitznagel et al., 2013, 2014; Alosco et al., 2014d).

All the studies investigating weight reduction in participants with obesity reported a general improvement in EF performances. Only Pearce et al. (2017) failed to detect any significant changes in performances.

Four studies assessed the effects of cognitive interventions on EFs in obese participants, showed a general improvement in executive performances associated with a reduction in body weight. Specifically, two studies evaluated the benefits of Cognitive Remediation Therapy (Alosco et al., 2014b; Allom et al., 2018), one assessed the impact of an intervention focused on Working Memory (Galioto et al., 2015) and one focused on the effects of a treatment aimed at strengthening cognitive functions in general (Verbeken et al., 2014).

Two further studies analyzed the trend over time of body weight and executive functioning in adults with obesity (Deckers et al., 2017; Stinson et al., 2018) without introducing weight reduction programs and reported inconsistent results. Deckers et al. (2017) found no relationship between weight changes and executive performance, while Stinson et al. (2018) found evidence of the role of EFs, specifically of reduced inhibitory control, in maintaining high body weight.

Eleven studies (Pauli-Pott et al., 2010; Spitznagel et al., 2013, 2014; Kulendran et al., 2014; Galioto et al., 2015, 2016; Xu et al., 2017; Augustijn et al., 2018; Dassen et al., 2018a; Stinson et al., 2018) investigated the predictive role of performance on executive tasks on body weight changes, and observed that appropriate executive functioning predicted a reduction in body weight in participants with obesity or overweight.

## Executive Functions in Longitudinal Studies

### Cognitive Flexibility

The tasks most commonly used to assess cognitive flexibility were the WCST, TMT, and Switching of Attention Task (see Table 4).

Of the eleven studies that investigated the relationship between cognitive flexibility and obesity (see Table 4), six (Bryan and Tiggemann, 2001; Alosco et al., 2014c; Spitznagel et al., 2014; Deckers et al., 2017; Augustijn et al., 2018; Stinson et al., 2018) failed to confirm this relationship. Those that found an association between obesity and executive functioning reported an improvement in performance as a result of weight reduction. Furthermore, negative performance appeared to be associated with less weight reduction over time (Spitznagel et al., 2013; Augustijn et al., 2018).

### Inhibition

The Stroop Color-Word Task and Stop-Signal Task were the tests most commonly used to investigate cognitive and motor inhibition (see Table 4).

Thirteen studies reported a relationship between BMI and cognitive inhibition (Bryan and Tiggemann, 2001; Pauli-Pott et al., 2010; Alosco et al., 2014d, 2015; Kulendran et al., 2014, 2017; Galioto et al., 2015, 2016; Xie et al., 2017; Xu et al., 2017; Augustijn et al., 2018; Stinson et al., 2018; Vantieghem et al., 2018). Some of these studies showed that inhibition control predicted a reduction of body weight considering both bariatric surgery (Kulendran et al., 2017) and weight loss programs (Pauli-Pott et al., 2010; Kulendran et al., 2014; Galioto et al., 2016; Xu et al., 2017; Augustijn et al., 2018; Stinson et al., 2018). Other studies showed an improvement in the inhibition after bariatric surgery (Alosco et al., 2014d, 2015; Galioto et al., 2015) or weight-loss programs (Bryan and Tiggemann, 2001; Xie et al., 2017; Vantieghem et al., 2018).

### Working Memory

The task most often used to investigate working memory was the Digit Span Test (Reynolds, 1997) (see Table 4).

Eight studies reported a negative relationship between working memory and body weight (Spitznagel et al., 2013, 2014; Alosco et al., 2014b,d, 2015; Galioto et al., 2015; Augustijn et al., 2018; Dassen et al., 2018b). Indeed some authors found an improvement of the performance in working memory tasks after bariatric surgery (Alosco et al., 2014b,d, 2015; Galioto et al., 2015), while other authors found a predictive role of working memory performance in the outcome of weight reduction programs (Augustijn et al., 2018; Dassen et al., 2018b) or bariatric surgery (Spitznagel et al., 2013, 2014); better performance predicted success of interventions. Conversely, three studies found no relationship between obesity and working memory (Bryan and Tiggemann, 2001; Galioto et al., 2016; Pearce et al., 2017).

### Decision-Making

Decision-making, as measured using the Iowa Gambling Task, did not appear to be directly associated with weight reduction in patients with obesity and overweight (Witbracht et al., 2012; Stinson et al., 2018). Only Demos et al. (2017) observed an improvement in decision-making following a reduction in body weight, but these authors used a task that employed food-related stimuli.

## Discussion

The results of the longitudinal studies confirmed the findings reported in cross-sectional studies, highlighting a relationship between executive functioning and overweight/obesity even if the direction of this relationship remains unclear.

Studies that analyzed the effects of treatments aimed at reducing body weight showed a general improvement in executive tasks as a result. This improvement appeared to occur both in adult populations (Bryan and Tiggemann, 2001; Witbracht et al., 2012) and in children and adolescents (Davis et al., 2011; Kulendran et al., 2014; Vantieghem et al., 2018). Moreover, studies focused on the ability of executive functioning to predict the success of weight-loss interventions found that higher executive functioning could be the cause of BMI reduction (see Table 4). Not all EFs appear to be related to obesity. Such inconsistency in the results could be due to different versions of the tasks used to evaluate EFs, as shown in the studies analyzed decision-making.

As for the cross-sectional studies, the authors interpreted the results based on two different types of theoretical models. One hypothesizes that excessive body weight is the cause of changes in executive functioning, according to results showing an improvement in executive tasks following treatment for weight loss (Davis et al., 2007a, 2011; Alosco et al., 2014a,b,c,d, 2015; Verbeken et al., 2014; Galioto et al., 2015; Demos et al., 2017; Xie et al., 2017; Vantieghem et al., 2018). In the other theoretical view, EFs are considered as predictors of eating behaviors related to excessive body weight, like overeating. Studies assessing the effects of strengthening EFs in participants with overweight or obesity (Verbeken et al., 2014; Allom et al., 2018; Dassen et al., 2018b; Raman et al., 2018) have observed both an increase in executive functioning and a reduction in BMI. This reduction may be due to improved eating behavior as a result of adequate working memory, cognitive flexibility, and inhibitory control. These enforcement functions would promote healthier behaviors, reducing the risk associated with obesity, and further improve weight reduction (Allom et al., 2018). Studies that have shown the predictive role of the EFs on the success of weight-loss treatments (Pauli-Pott et al., 2010; Spitznagel et al., 2013, 2014; Kulendran et al., 2014, 2017; Galioto et al., 2016; Xu et al., 2017; Augustijn et al., 2018) confirmed the critical role of executive functioning in the occurrence of obesity.

Concerning bariatric surgery, the effects of weight-loss on executive performances resulted only at the follow-up (Spitznagel et al., 2013; Alosco et al., 2014a; Pearce et al., 2017). This result could be interpreted in two ways. On the one hand, it may suggest that a reduction in body fat favors improvement in executive functioning (Alosco et al., 2014b) as a consequence of the resolution of metabolic alterations related to excessive BMI; on the other hand, better performance at baseline could lead to an improvement in healthy eating habits (Spitznagel et al., 2013; Pearce et al., 2017), linked to a reduction of BMI over time. This last interpretation is supported by the results observed at the follow-up that showed a higher reduction in BMI in participants presenting better EFs performance at baseline (Spitznagel et al., 2013; Pearce et al., 2017). Lastly, it is interesting to note that control groups with obesity that did not benefit from the treatments (Bryan and Tiggemann, 2001; Alosco et al., 2014c; Pearce et al., 2017; Xie et al., 2017) did not show improvement in performance on cognitive tasks in the follow up assessment. These results confirm that a reduction in body fat leads to gains in executive functioning, although the groups that did not benefit from the treatment did not show a further executive decline.

Despite these findings, short-term follow-ups showed no evidence of a causal relationship of EFs on obesity. These studies did not observe significant differences between participants with obesity who have reduced their body weight and those who maintained their condition unchanged (Deckers et al., 2017). In line with these results, we can conclude that the relationship between EFs and excessive body weight appears robust even when longitudinal studies are considered. However, even considering the results of longitudinal studies appear challenging to determine the direction of this relationship, and further studies are needed.

## General Discussion

Only in recent years, the studies focused their attention on the relationship between excessive body weight and EFs (Fitzpatrick et al., 2013). This relationship appears to be confirmed by most of the studies, both cross-sectional (e.g., Verdejo-García et al., 2010; Cohen et al., 2011; Maayan et al., 2011; Dassen et al., 2018a) and longitudinal (e.g., Spitznagel et al., 2013; Alosco et al., 2014d, 2015; Augustijn et al., 2018), analyzed in this systematic review, despite the heterogeneity of the tasks used and the methodological framework adopted. Functional and neuroimaging studies confirmed changes in the cortical areas involved in executive functioning in participants with obesity (Stingl et al., 2012; Alarcón et al., 2016; Tsai et al., 2016) even when cognitive tasks failed to highlight any significant differences in performance between obesity and normal-weight conditions (Hendrick et al., 2012; Frank et al., 2014; Pearce et al., 2017).

The choice to selected studies which considered different aged made us possible to highlight a similar pattern in the relationship between EFs and overweight/obesity in children (Blanco-Gómez et al., 2015; Tsai et al., 2016) and adults (Cohen et al., 2011; Deckers et al., 2017), despite the individual differences linked to age.

This systematic review allowed us to observe poor performance on executive function tasks also in people with overweight, not only in those with obesity (Verdejo-García et al., 2010; Sellaro and Colzato, 2017), although only a few studies have investigated the condition of overweight (BMI between 25 and 30) compared to normal-weight (BMI lower than 25) and obesity (BMI higher than 30). These results should be explored in further studies to verify how executive functioning is expressed at the different stages of overweight and to understand if the early intervention could prevent the worsening of the increase in adiposity.

As previously reported, the results of these studies have been interpreted according to two different theoretical models. At the conclusion of this systematic review, no single theoretical model appears to prevail. The empirical data seem to support both theoretical models: the one postulating the influence of executive system dysfunctions on obesity (Drewnowski, 1997; Goldstone et al., 2009; Smith and Robbins, 2013), the other viewing impairment of executive functioning as a consequence of the obesity (Ricca et al., 2009; Pieper and Laugero, 2013).

Other longitudinal studies are needed to disentangle the relationship between obesity and executive dysfunctions. These studies could either examine the eating behavior and BMI of people with low executive functioning over time or monitor the executive functionality of people with overweight who become obese over time. Finally, the possibility that the relationship between executive dysfunctions and overweight/obesity could be bidirectional cannot be excluded; in fact, many studies seem to suggest that the bidirectionality is the real nature of this relation (Spitznagel et al., 2013; Augustijn et al., 2018; Raman et al., 2018).

This systematic review has some strengths, such as the decision to exclude studies of children of preschool age (younger than 5 years) and those over 70 years. This decision was taken for various reasons. First, the EFs and underlying neural areas of preschool-age children are immature and still developing (Diamond, 2013); moreover, during this period children are still introjecting eating habits learned from the external environment (Guxens et al., 2009; Gregory et al., 2010). However, previous studies have shown a specific predictive value of EFs performance in preschool children concerning weight and eating behaviors (Park et al., 2014), and for this reason, it would be interesting to study this specific age group separately. The decision to not include studies of people over 70 years of age was influenced by the “obesity paradox” hypothesis (Artham et al., 2008; Park et al., 2014), which recognizes the health benefits to older people in having a higher BMI. Furthermore, impairment of EFs in older people can be associated with the aging process (Fjell et al., 2016). Although analysis of the relationship between EFs and overweight/obesity in these two age groups could be interesting, their inclusion in the present study would have led to extreme heterogeneity.

## Limitations

This systematic review was not able to identify if one specific EF had a more significant role than another on the analyzed relationship. This result could represent a limitation of this study because it has not allowed us to establish whether differences in performance were due to changes in some functions rather than others. This limitation is due mainly to the heterogeneity of cognitive tasks (Yang et al., 2018). Another limit could be represented by the selection of participants from 5–70 years; in fact, also if the results are coherent, it is known that the brain continues to develop from childhood to young adulthood, and the differences related to aging could influence the relation between cognitive aspects and weight changes. These age-related differences may have covered possible results that could indicate a causal direction between the variables.

Considering the longitudinal studies, the most extended follow-up period—of 4 years—was performed by Alosco et al. (2014d), though with considerable data loss. No other study investigated the relationship between EFs and body weight following body loss treatment over such a long time. This aspect represents a further limitation of the results, i.e., it is not clear whether the improvements were sustained over time or whether a subsequent reversal of the trend occurs, which might have been the reason behind the drop-out from treatment among bariatric patients. Besides, a possible change in the trend over time could indicate that it is the executive damage that influences the success of weight-loss interventions.

Another limitation is represented by not having included in the systematic review the analysis of psychological variables that could modulate the relationship between EFs and excessive body weight. In fact, a few of the selected studies controlled the psychological dimensions, like anxiety, depression, or emotional regulation. It would be interesting to carry out an analysis of these dimensions. One of the studies that considered some emotional components showed that emotions could modulate the relationship between executive functioning and obesity. However, others found no effect of psychological variables on this relationship (Yau et al., 2014). Even so, examining these psychological variables might also lead to a better understanding of the Emotionally-Driven Eating Model (Chen et al., 2017) in individuals with no eating disorders.

Further limitations are due to the limited samples considered by both cross-sectional and longitudinal studies and by the higher prevalence of females among the participants that do not allow to generalize the results. Weller et al. (2008) found different results between males and females on EF performances, and further studies would be useful to analyse gender differences.

A significant limitation of this work concerns the lack of studies comparing participants with overweight and obesity separately. This comparison would have allowed us to examine the relationship between different severities of excessive body weight and the impairment of executive functioning, and to identify the nature of this relationship. Furthermore, a specific focus on participants with overweight would also have led to determine the cognitive characteristics that might serve as warning signs of the development of obesity.

Finally, a meta-analysis measuring the statistical power of the results obtained from the studies analyzed in this study could help to interpret better the results obtained in this systematic review.

## Conclusions

The analysis of studies on the relationship between executive functioning and excessive body weight did not give us decisive responses to all the questions advanced by this systematic review but clarified a large part of the issues on this topic. A consistent relationship between executive functioning and overweight/obesity has been confirmed, but it remains unclear whether a general executive dysfunction is involved or whether one EF is more implicated than others.

Although it was not possible to confirm a specific theoretical model on the relationship between EFs and overweight/obesity, the association between these dimensions appears to be the result of a complex interaction between different factors that influence both people's attitude to food and eating and their executive functioning. Prolonged inappropriate food intake related to the maintenance of excessive body weight leads to poorer performance on executive tasks. Furthermore, executive impairment exacerbates inappropriate behaviors, leading to increased body fat (Stinson et al., 2018). Both these aspects are associated with a real risk of cognitive impairment in old age (Sanderlin et al., 2017) and difficulty in responding appropriately to external stimuli that is typical of executive dysfunctions and which would negatively affect the life of obese individuals. It is essential to intervene in both these dimensions to reduce the impact of obesity on quality of life.

It would be interesting in future to evaluate the effectiveness of long-term interventions involving weight-loss programmes. The success of weight-loss interventions may be strictly linked to an improvement in executive functioning because effective executive skills would allow healthier lifestyles. In this context, it might be useful to examine the integrated model of Sellbom and Gunstad (2012) and the Emotionally-Driven Eating model (Gianini et al., 2013; Wagner et al., 2013) in terms of the relationship between BMI and cognitive functioning variables such as mood and emotional regulation that were not often analyzed in the studies reviewed here.

The leading role of this systematic review was to underline the powerful connection between cognitive aspects, specifically EFs, and excessive body weight, highlighting the importance of considering the nature of the link between these variables in studies on overeating and obesity. A relevant suggestion that emerges by this study is the need for longitudinal studies which, starting from the analysis of EFs, monitor the BMI over time.

It could be essential for structuring intervention aimed at enhancing EFs to prevent the drop-out rate among patients with severe obesity who fail to benefit for a long time from the effects of treatments (Galioto et al., 2016; Xu et al., 2017), by favoring the long-term maintenance of the lower weight achieved. It needs to reduce the risk of further weight gain in people with overweight, thereby preventing the occurrence of severe obesity. Moreover, an integrated approach that also takes emotion regulation and mood into account could be the best strategy for countering dysfunctional eating behaviors and executive functioning; therefore it would be necessary to develop an integrated theoretical model that should jointly consider EFs, eating behavior, emotion regulation, and mood in the field of overweight and obesity.

## Author Contributions

MC and FF conception of the review, the literature search, and the writing of the manuscript. GF resolved disagreements in the choice of the articles and also contributed to the discussions of the analyzed searches and the revisions of the manuscript. The authors revised, read, and approved the submitted version.

### Conflict of Interest Statement

The authors declare that the research was conducted in the absence of any commercial or financial relationships that could be construed as a potential conflict of interest.
